# Association between life’s essential 8 and activities of daily living disability in Chinese adults aged 80 years and older: a cross-sectional study from the CLHLS

**DOI:** 10.3389/fragi.2026.1845642

**Published:** 2026-07-10

**Authors:** Jiaxin Huang, Bing Feng, Ge Chang, Chaoqun Geng, Weimin Bai, Jianchao Li

**Affiliations:** 1 Department of Extracorporeal circulation, Fuwai Central China Cardiovascular Hospital, Henan Cardiovascular Disease Center (Central China Subcenter of National Center for Cardiovascular Diseases), Zhengzhou, China; 2 Department of Traditional Chinese Medicine Rehabilitation, The 966th Hospital of the PLA Joint Logistic Support Force, Dandong, China; 3 Key Laboratory of Medical Laboratory, Henan Provincial Chest Hospital, Affiliated Chest Hospital of Zhengzhou University, Zhengzhou, China; 4 Department of Emergency, Henan Provincial People’s Hospital, People’s Hospital of Zhengzhou University, People’s Hospital of Henan University, Zhengzhou, China

**Keywords:** activities of daily living disability, cardiovascular health, functional independence, healthy ageing, life’s essential 8, oldest-old

## Abstract

**Background:**

Maintaining functional independence is a major goal of healthy ageing in the oldest-old, yet evidence on the association between Life’s Essential 8 (LE8)and activities of daily living (ADL) disability in this population remains limited. We examined the associations between LE8 and ADL disability among Chinese adults aged ≥80 years.

**Methods:**

This cross-sectional studyincluded 909 adults aged ≥80 years from the 2014 wave of the Chinese Longitudinal Healthy Longevity Survey biomarker sub-study. Cardiovascular health was assessed using the American Heart Association’s LE8framework. ADL disability was defined as at least one limitation across six basic activities, and disability severity was categorized as 0, 1, or ≥2 limitations. Multivariable logistic regression and proportional odds ordinal logistic regression were used to examine associations of LE8 with ADL disability and disability severity.

**Results:**

In the fully adjusted model, each 10-point higher LE8 score was associated with reduced odds of ADL disability [odds ratio (OR): 0.77, 95% confidence interval (CI): 0.62–0.95], and participants in the highest tertile had reduced odds than those in the lowest tertile (OR: 0.57, 95% CI: 0.34–0.95). Similar associations were observed for disability severity (per 10-point increase: OR: 0.77, 95% CI: 0.62–0.94). Restricted cubic spline analysis showed a significant overall association without strong evidence of nonlinearity. In component-level analyses, physical activity and diet showed the most prominent inverse associations with ADL disability.

**Conclusion:**

Higher LE8 scores were associated with a lower ADL disability burden among Chinese adults aged ≥80 years. These fingdings support the potential value of LE8 as an integrated framework for characterizing functional vulnerability in the oldest-old.

## Introduction

Population ageing is accelerating globally, and China is undergoing one of the most pronounced demographic transitions toward advanced age (Chen et al., 2022). Disability in basic activities of daily living (ADL) is common among the oldest-old and is closely related to loss of independence, institutionalization, caregiver burden, and increased healthcare expenditure (Hou et al., 2018; Wang Z. et al., 2023; Enroth et al., 2021; Chen et al., 2019). In this population, functional status is a particularly important outcome, as it reflects the combined effects of disease burden, physiologic reserve, and the capacity for independent living.

Cardiovascular and metabolic health are increasingly recognized as important determinants of functional status in later life. Lifestyle-related factors, including diet, physical activity, smoking exposure, and sleep, as well as biological factors such as adiposity, blood pressure, glycemic status, and lipid profiles, have been linked to sarcopenia, frailty, cerebrovascular injury, systemic inflammation, and functional decline (Millan-Domingo et al., 2024; Veler, 2023; Rouch et al., 2022; Kitamura et al., 2021; Tang et al., 2023). However, these factors are often examined separately, which may underestimate their combined relevance to functional ageing. Accordingly, an integrated framework that captures multiple modifiable dimensions of cardiovascular health may be useful for characterizing functional vulnerability in older adults.

The American Heart Association (AHA) introduced Life’s Essential 8 (LE8) as an updated construct of cardiovascular health, integrating eight modifiable components, diet, physical activity, nicotine exposure, sleep health, body mass index (BMI), blood lipids, blood glucose, and blood pressure, into a standardized scoring system ranging from 0 to 100 (Lloyd-Jones et al., 2022). Accumulating evidence indicates that higher LE8 scores are associated with lower risks of major cardiovascular events, mortality, and other adverse health outcomes in both general adult and older populations (Sun et al., 2023; Wu et al., 2023). However, evidence specifically linking LE8 to functional outcomes, particularly ADL disability, remains limited (Gómez-Ángel et al., 2025; Li et al., 2025; Chen et al., 2024).

Several knowledge gaps remain in the existing literature. First, prior studies have largely focused on middle-aged individuals or younger older adults, with relatively limited evidence among the oldest-old (Jerome and Lilly, 2024). Second, functional outcomes have often been assessed using heterogeneous measures, including mobility limitations, self-reported functioning, or composite frailty indices, thereby complicating interpretation with respect to ADL disability specifically (Gómez-Ángel et al., 2025). Third, although LE8 provides an integrated cardiovascular health framework, the relative associations of its individual components with ADL disability in advanced age remain insufficiently understood (Li et al., 2025).

Focusing on the oldest-old is particularly important, as exposure–outcome relationships may differ from those observed in younger populations. Survivorship bias, multimorbidity, polypharmacy, and age-related physiologic changes may modify the associations between traditional risk factors, including blood pressure and BMI, and late-life functional outcomes, and thresholds considered optimal in younger adults may not be directly applicable in advanced age (Wang et al., 2018; Satish et al., 2001). Moreover, ADL disability in this population often reflects the cumulative effects of vascular, metabolic, musculoskeletal, and neurocognitive processes, underscoring the need for integrated approaches to risk assessment within the context of healthy ageing. These features highlight the need to examine whether LE8 is associated with functional disability specifically in very old adults.

Using data from the Chinese Longitudinal Healthy Longevity Survey (CLHLS), the present study examined the associations between LE8 and ADL disability among Chinese adults aged 80 years and older. We hypothesized that higher LE8 scores would be associated with lower odds of ADL disability and lower disability severity in this oldest-old population. This study extends previous research by focusing specifically on Chinese adults aged ≥80 years, evaluating both binary ADL disability and ordinal disability severity, and exploring the associations of individual LE8 components with ADL disability.

## Materials and methods

### Study design and sample

This study utilized data from the CLHLS, an ongoing nationwide longitudinal cohort initiated in 1998 with follow-up surveys conducted approximately every 2–3 years. The CLHLS was implemented in randomly selected counties and cities across 22 of 30 provinces, representing approximately 85% of the Chinese population. Detailed descriptions of the sampling strategy and data quality assurance procedures have been reported previously (Song et al., 2021). The study protocol was approved by the Research Ethics Committees of Peking University and Duke University (IRB00001052-13074), and written informed consent was obtained from all participants or their legal representatives.

This cross-sectional analysis was based on the 2014CLHLS biomarker sub-study, which was conducted in selected longevity areas across eight provinces. Of 7,192 individuals interviewed in the 2014 CLHLS, 2,542 participated in a biomarker sub-study and provided blood samples. Because calculation of LE8 in the present study required biomarker data, the analysis was restricted *a priori* to this biomarker sub-cohort. Among these individuals, 1,726 were aged ≥80 years. After excluding participants with incomplete data required to construct LE8 and those with missing ADL data, 909 participants were included in the final analytic sample. This study was reported in accordance with the STROBE guidelines (von Elm et al., 2008).

### Assessment of cardiovascular health (life’s essential 8)

Cardiovascular health was assessed using the AHA LE8 framework, which comprises eight components: diet, physical activity, nicotine exposure, sleep health, BMI, blood lipids, blood glucose, and blood pressure (Lloyd-Jones et al., 2022). Each component was scored from 0 to 100, with higher scores indicating more favorable cardiovascular health, and the overall LE8 score was calculated as the mean of the eight component scores.

Where possible, LE8 components were operationalized according to the original AHA definitions. However, because the CLHLS did not collect all information required for the original LE8 algorithm, the diet, physical activity, and blood glucose components were adapted based on available data. Diet and physical activity scores were constructed using available questionnaire items following previously published CLHLS-based approaches and were linearly transformed to a 0–100 scale to align with the AHA LE8 scoring framework (Wang J. et al., 2023). Detailed questionnaire items, component definitions, scoring criteria, and original component distributions are provided in the Supplementary Material.

For the blood glucose component, HbA1c data were not available in the CLHLS. Therefore, fasting plasma glucose was used to derive an HbA1c-equivalent value based on a published conversion equation, and this derived value was used to assign the LE8 glucose score according to AHA categories (Nathan et al., 2008). The formula and detailed scoring procedure are described in the Supplementary Material.

### Assessment of ADL disability

ADL disability was assessed using six basic activities: bathing, dressing, toileting, indoor transferring, continence, and eating. Participants were asked: “For at least the last 6 months, have you been limited in activities people usually do because of a health problem?” for each domain. Response options were categorized as without assistance, one-part assistance, or more-than-one-part assistance. For each ADL item, any response indicating the need for assistance (one-part or more-than-one-part assistance) was classified as a limitation.

Participants were classified as having ADL disability if they reported ≥1 limitation across the six domains. For severity analyses, ADL disability was categorized into three levels: 0 limitations, 1 limitation, and ≥2 limitations, with ≥2 limitations considered severe disability.

### Covariates

Covariates were selected *a priori* based on clinical relevance and prior literature, including age, sex, residence (rural vs. urban), marital status (unmarried [never married, widowed, or divorced] vs. married), economic status (poor, fair, or rich), self-rated health (very good, good, fair, poor, or very poor), and education (>5 years vs. ≤ 5 years). In additional analyses, comorbidity burden was summarized as the number of chronic conditions, including hypertension, diabetes, heart disease, and stroke/cerebrovascular disease. Co-residence status was also considered in sensitivity analyses to account for whether participants lived alone or with family members or others.

### Statistical analysis

Baseline characteristics were summarized across LE8 tertiles and compared using analysis of variance (ANOVA) for continuous variables and the χ^2^ test for categorical variables.

Associations between LE8 and ADL disability were evaluated using logistic regression for the binary outcome and proportional odds ordinal logistic regression for the ordinal outcome, with LE8 modeled as tertiles and per 10-point increment. Three prespecified models were fitted: Model 1, unadjusted; Model 2, adjusted for age and sex; and Model 3, further adjusted for economic status, education, residence, self-rated health, and marital status.

Restricted cubic spline analyses were used to explore potential nonlinearity in the association between LE8 and ADL disability. Component-level analyses were conducted to examine the associations of individual LE8 components with ADL disability. Prespecified subgroup analyses were performed by sex, age group, chronic disease burden, and frailty status, with interaction terms used to assess potential effect modification. Sensitivity analyses were conducted to assess the robustness of the findings, including additional adjustment for comorbidity burden and co-residence status, exclusion of participants with poor or very poor self-rated health, exclusion of bedridden participants, exclusion of participants with pre-existing heart disease or stroke/cerebrovascular disease, and use of severe ADL disability as an alternative outcome definition. Detailed procedures for spline analyses, component-level analyses, subgroup analyses, sensitivity analyses, and model diagnostics are provided in the Supplementary Statistical Methods.

All statistical tests were two-sided, with P < 0.05 considered statistically significant. Analyses were conducted using Stata version 16.0 (StataCorp, College Station, TX, United States) and R version 3.6.1 (R Foundation for Statistical Computing, Vienna, Austria).

## Results

### Baseline characteristics across LE8 tertiles

A total of 909 participants were included in the final analytic sample (Supplementary Figure S1). Baseline characteristics stratified by LE8 tertiles are presented in Table 1. The mean age was 90.78 years, and 59.5% of participants were women. Participants in the highest LE8 tertile were younger, more likely to reside in urban areas, and differed in economic status compared with those in the lower tertiles. Higher LE8 tertiles were also associated with a lower comorbidity burden, as reflected by a lower mean number of chronic conditions and reduced prevalences of hypertension and diabetes (all *P* < 0.001). Overall, 14.5% of participants had ADL disability and 7.2% had severe ADL disability. The prevalence of ADL disability decreased across LE8 tertiles (18.2%, 15.7%, and 9.5%; *P* = 0.009), with a similar trend observed for severe disability (*P* = 0.039). In addition, the original distributions of the eight individual LE8 component variables before score transformation are provided in Supplementary Table S3, while the distribution of overall LE8 scores and ADL limitation counts are shown in Supplementary Figure S2 and Supplementary Table S4, respectively. Comparisons between included and excluded participants are presented in Supplementary Table S5.

**TABLE 1:** Baseline characteristics of participants aged ≥80 years by LE8 tertiles (N = 909).

Characteristic	Overall (N = 909)	Tertile 1 (n = 303)	Tertile 2 (n = 312)	Tertile 3 (n = 294)	P value
Age, years	90.78 (7.64)	91.02 (7.51)	91.43 (7.73)	89.84 (7.62)	0.030
Female, n (%)	541 (59.5)	187 (61.7)	187 (59.9)	167 (56.8)	0.465
Education >5 years, n (%)	72 (7.9)	29 (9.6)	23 (7.4)	20 (6.8)	0.414
Urban residence, n (%)	188 (20.7)	50 (16.5)	63 (20.2)	75 (25.5)	0.024
Married, n (%)	254 (27.9)	75 (24.8)	88 (28.2)	91 (31.0)	0.239
Economic status, n (%)					
Poor	72 (7.9)	38 (12.5)	20 (6.4)	14 (4.8)	0.004
Fair	695 (76.5)	225 (74.3)	243 (77.9)	227 (77.2)	​
Rich	142 (15.6)	40 (13.2)	49 (15.7)	53 (18.0)	​
Self-rated health, n (%)	​	​	​	​	0.001
Good/very good	466 (51.3)	137 (45.2)	156 (50.0)	173 (58.8)	​
Fair	356 (39.1)	127 (41.9)	122 (39.1)	107 (36.4)	​
Poor/very poor	87 (9.6)	39 (12.9)	34 (10.9)	14 (4.8)	​
Chronic disease					
Hypertension, n (%)	292 (32.1)	139 (45.9)	100 (32.1)	53 (18.0)	<0.001
Heart disease, n (%)	95 (10.5)	40 (13.2)	25 (8.0)	30 (10.2)	0.108
Diabetes, n (%)	42 (4.6)	31 (10.2)	7 (2.2)	4 (1.4)	<0.001
Stroke/cerebrovascular disease, n (%)	57 (6.3)	23 (7.6)	10 (3.2)	24 (8.2)	0.022
Number of chronic conditions, mean (SD)	0.53 (0.70)	0.77 (0.77)	0.46 (0.63)	0.38 (0.64)	<0.001
LE8 total score, mean (SD)	70.54 (9.61)	60.02 (5.52)	70.81 (2.53)	81.10 (4.62)	<0.001
ADL disability (≥1 limitation), n (%)	132 (14.5)	55 (18.2)	49 (15.7)	28 (9.5)	0.009
Severe ADL disability (≥2 limitations), n (%)	65 (7.2)	28 (9.2)	25 (8.0)	12 (4.1)	0.039
ADL limitations, count, mean (SD)	0.37 (1.13)	0.46 (1.22)	0.43 (1.28)	0.21 (0.81)	0.012

### Association between LE8 and ADL disability

As shown in Table 2, higher LE8 scores were consistently associated with lower odds of ADL disability among participants aged ≥80 years. For the binary outcome, each 10-point increment in LE8 was associated with a 23% reduction in the odds of disability in the fully adjusted model (OR: 0.77, 95% CI: 0.62–0.95, *P* = 0.013). When LE8 was categorized into tertiles, participants in the highest tertile exhibited significantly lower odds of ADL disability compared with those in the lowest tertile (OR: 0.57, 95% CI: 0.34–0.95, *P* = 0.031).

**TABLE 2:** Association of LE8 with ADL disability (binary) and ADL disability severity (ordinal) among participants aged ≥80 years (N = 909).

Outcome/Exposure	Model 1 OR (95% CI)	P	Model 2 OR (95% CI)	P	Model 3 OR (95% CI)	P
Outcome: ADL disability vs. no disability (binary logistic regression)						
LE8 (per +10 points)	0.73 (0.61–0.88)	<0.001	0.74 (0.61–0.90)	0.002	0.77 (0.62–0.95)	0.013
LE8 tertiles: Middle vs. worst	0.84 (0.55–1.28)	0.419	0.80 (0.52–1.25)	0.327	0.80 (0.51–1.25)	0.331
LE8 tertiles: Best vs. worst	0.47 (0.29–0.77)	0.003	0.51 (0.31–0.84)	0.009	0.57 (0.34–0.95)	0.031
Outcome: ADL disability severity (ordinal logistic regression)						
LE8 (per +10 points)	0.73 (0.60–0.88)	0.001	0.73 (0.60–0.90)	0.003	0.77 (0.62–0.94)	0.012
LE8 tertiles: Middle vs. worst	0.84 (0.55–1.28)	0.420	0.80 (0.52–1.24)	0.322	0.79 (0.51–1.23)	0.298
LE8 tertiles: Best vs. worst	0.47 (0.29–0.77)	0.002	0.50 (0.30–0.83)	0.007	0.57 (0.34–0.94)	0.028

Model 1: crude;

Model 2: adjusted for age and sex;

Model 3: adjusted for age, sex, education, residence, marital status, self-rated health and economic status.

Proportional odds assumption (LR, test): *P* all >0.05.

Consistent findings were observed when ADL disability severity was analyzed as an ordinal outcome. Each 10-point increment in LE8 was associated with lower odds of being in a more severe disability category (OR: 0.77, 95% CI: 0.62–0.94, *P* = 0.012), and participants in the highest tertile had significantly lower disability severity than those in the lowest tertile (OR: 0.57, 95% CI: 0.34–0.94, *P* = 0.028). Model-based predicted probability curves are presented in the Supplementary Figure S3.

**FIGURE 1:**
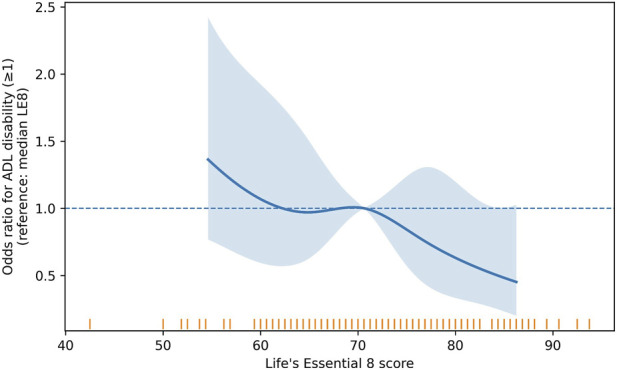
Restricted cubic spline association between Life’s Essential 8 (LE8) score and ADL disability. The curve depicts the multivariable-adjusted odds ratio (OR) and 95% confidence interval (CI) for ADL disability (≥1 limitation) across the observed range of LE8 scores using restricted cubic splines with five knots (*P*-overall = 0.041, *P*-nonlinear = 0.960) The reference value was set at LE8 = 70 (median valus, as specified in the figure). Estimates were derived from Model 3.

The RCS analysis of continuous LE8 in relation to ADL disability is presented in Figure 1. The overall association was statistically significant (*P* for overall = 0.041), with no evidence of nonlinearity (*P* for nonlinearity = 0.096).

### Component-level analyses

**FIGURE 2:**
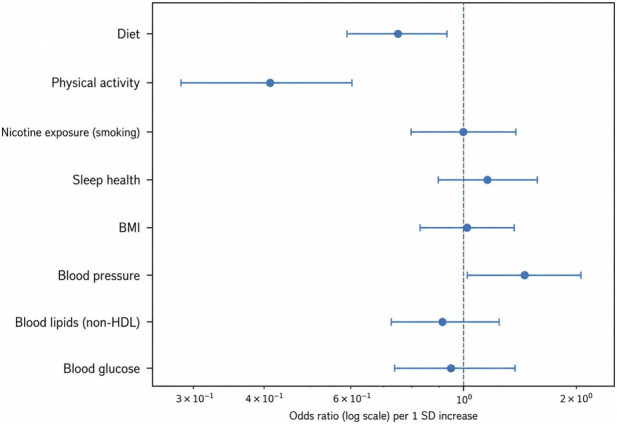
Associations between individual LE8 component scores and ADL disability. Forest plot showing adjusted ORs (95% CIs) for ADL disability (≥1 limitation) per 1-standard deviation (SD) increase in each LE8 component score (nicotine exposure, sleep health, body mass index, blood pressure, physical activity, diet, blood lipids, and blood glucose). Estimates were derived from Model 3.

Component-level associations (per 1-SD increase in each LE8 component score) are presented in Figure 2 and Supplementary Table S6. In the fully adjusted model, physical activity demonstrated the strongest inverse association with ADL disability (OR: 0.31, 95% CI: 0.22–0.44, *P* < 0.001), and diet was also inversely associated with the outcome (OR: 0.78, 95% CI: 0.64–0.95, *P* = 0.013). In contrast, the blood pressure component score was positively associated with ADL disability (OR: 1.22, 95% CI: 1.01–1.46, *P* = 0.039), whereas the remaining components were not materially associated with the outcome.

Given the unexpected direction of the blood pressure component, additional exploratory analyses were conducted using original systolic and diastolic blood pressure values and by recalculating the overall LE8 score after removing the blood pressure component. These results are presented in the Supplementary Figures S4-S6 and Supplementary Tables S7, S8. After excluding the blood pressure component, the association between overall LE8 and ADL disability appeared stronger than that observed in the primary analysis (OR per 10-point increase: 0.68 vs. 0.77), suggesting that the blood pressure component may have attenuated the overall association.

### Subgroup analyses

Subgroup findings are presented in Supplementary Table S9. The inverse association between LE8 (per 10-point increment) and ADL disability was generally consistent across subgroups defined by sex, age group, chronic disease burden, and frailty status based on the frailty index, with no evidence of effect modification (all *P* for interaction >0.10).

### Sensitivity analyses

Sensitivity analyses yielded results that were broadly consistent with the primary findings. Similar patterns were observed when severe ADL disability (≥2 limitations) was used as an alternative outcome, after additional adjustment for the number of chronic conditions, after excluding participants with poor or very poor self-rated health, after excluding those with pre-existing heart disease or stroke/cerebrovascular disease, after excluding bedridden participants, and after additional adjustment for co-residence status. The direction and magnitude of the associations remained comparable to those observed in the primary analyses (Supplementary Tables S10–S15).

In additional sensitivity analyses focused on physical activity, the inverse association between the physical activity component and ADL disability remained statistically significant after excluding participants with poor or very poor self-rated health and after excluding bedridden participants, suggesting that this association was not solely driven by individuals with markedly poor perceived health or severe functional restriction (Supplementary Tables S16,S17).

## Discussion

In this cross-sectional study of Chinese adults aged ≥80 years,we examined the associations between LE8 and ADL disability. Consistent with our hypothesis, higher LE8 scores were associated with lower odds of ADL disability and lower disability severity. The association was observed in both continuous and tertile-based analyses and was generally supported by spline, subgroup, and sensitivity analyses. At the component level, physical activity showed the strongest inverse association with ADL disability, and diet was also inversely associated with the outcome. These findings suggest that LE8 may serve as an integrated framework for characterizing functional vulnerability in the oldest-old.

Our findings are broadly aligned with a growing body of evidence linking higher LE8 scores to more favorable functional outcomes in older populations. In the Seniors-ENRICA-2 cohort, higher LE8 scores were associated with both lower prevalence and reduced incidence of impaired lower-extremity function, with physical activity, glucose, BMI, and nicotine exposure contributing substantially to these associations (Gómez-Ángel et al., 2025). Similarly, in the InCHIANTI study, higher LE8 scores were associated with lower risks of both ADL and IADL disability, as well as reduced functional decline over time (Li et al., 2025). Furthermore, a cross-sectional analysis of NHANES data among adults with osteoarthritis demonstrated that higher LE8 scores were associated with a lower prevalence of disability across multiple functional domains (Chen et al., 2024). The present study extends this literature by demonstrating that higher LE8 scores are associated with both lower odds of ADL disability and reduced disability severity specifically among Chinese adults aged ≥80 years. This contribution is relevant because the oldest-old remain underrepresented in LE8-related research, although ADL disability is a key indicator of loss of independence in this age group. In addition, our component-level analyses provide preliminary evidence regarding which LE8 components may be most closely related to functional disability in very old adults.

The association between higher LE8 scores and reduced odds of disability is biologically plausible, particularly in the context of advanced age. In the oldest-old, ADL disability rarely arises from a single pathological process; rather, it typically reflects the cumulative burden of vascular injury, metabolic dysregulation, chronic inflammation, sarcopenia, frailty, and neurocognitive decline (Kitamura et al., 2021; Andonian et al., 2025; Libon et al., 2026). Because LE8 integrates multiple modifiable behavioral and biological dimensions of health, it may provide a more comprehensive representation of the multifactorial processes underlying functional decline in late life (Lloyd-Jones et al., 2022). Therefore, higher LE8 scores may reflect more favorable overall health and greater physiological reserve, which are important for maintaining mobility, self-care capacity, and independent living in advanced age (Xu and Zhang, 2025; Zhao and Fan, 2025).

Among the individual LE8 components, physical activity demonstrated the strongest inverse association with ADL disability, which is biologically consistent with its established contributions to muscle strength, balance, mobility, cardiopulmonary fitness, and overall physiologic reserve (Bilski et al., 2022; Thomas et al., 2019; Álvarez-Bustos et al., 2025; Jarvie et al., 2019; Yin et al., 2025). Diet was also inversely associated with disability, suggesting that nutritional quality may play a key role in maintaining muscle mass, metabolic stability, and physiologic resilience in late life (Millan-Domingo et al., 2024; Ni et al., 2021; Matsuyama et al., 2022). These findings suggest that lifestyle-related LE8 components may be particularly relevant to functional independence in the oldest-old. Nevertheless, the strong association observed for physical activity should be interpreted with appropriate caution, as reverse causation cannot be fully excluded in a cross-sectional design. Older adults with ADL limitation may reduce their physical activity because of existing functional impairment. To address this concern, we conducted sensitivity analyses excluding participants with poor or very poor self-rated health and excluding bedridden participants. The inverse association between physical activity and ADL disability remained generally consistent in these analyses, suggesting that the finding was not solely driven by participants with markedly poor perceived health or severe functional restriction. In contrast, the remaining LE8 components were not materially associated with disability, which may reflect limited statistical power for component-specific analyses, measurement error related to adapted operational definitions, survivor bias, and the possibility that certain biological risk factors are less closely linked to contemporaneous functional status than behavioral factors in this oldest-old population.

The blood pressure component exhibited a pattern that differed from most other LE8 components. Although the standard LE8 framework assumes that lower blood pressure reflects more favorable cardiovascular health, the present findings suggest that this assumption may not translate directly to functional outcomes in the oldest-old. Specifically, the blood pressure component score was associated with ADL disability in an unexpected direction. In exploratory analyses, the association between overall LE8 and ADL disability appeared stronger after removing the blood pressure component, suggesting that this component may have attenuated the overall LE8–ADL association. These observations suggest that the current AHA blood pressure scoring framework may not fully capture the functional implications of blood pressure in very old adults. This interpretation is supported by prior evidence indicating that lower systolic blood pressure is not consistently associated with improved outcomes in this age group. For example, in the CLHLS cohort, systolic blood pressure demonstrated a U-shaped association with 3 year all-cause mortality, with the lowest risk observed in the mid-range (107–154 mmHg) rather than at lower levels (Lv et al., 2018). Similarly, in routine primary care settings in England, adults aged ≥80 years with treated hypertension exhibited higher mortality when achieved systolic blood pressure was <135 mmHg compared with 145–154 mmHg (Delgado et al., 2017). In the Leiden 85-plus study, lower systolic blood pressure among individuals receiving antihypertensive therapy was also associated with increased mortality and more rapid cognitive decline (Streit et al., 2018). Taken together, these findings suggest that lower blood pressure in very old adults may sometimes reflect frailty, multimorbidity, reduced physiological reserve, or treatment-related vulnerability rather than optimal health status. Therefore, the blood pressure component should be interpreted cautiously when applying LE8 to disability-related outcomes in the oldest-old.

This study has several notable strengths. It specifically targeted the oldest-old, a population that remains underrepresented in research examining LE8 and functional outcomes, and evaluated both ADL disability and disability severity, which are critical indicators of functional independence in late life. In addition to analyzing the overall LE8 score, the study further examined individual components and incorporated multiple sensitivity analyses that consistently supported the robustness of the primary findings. Several limitations should also be considered. First, the cross-sectional design precludes causal inference and does not allow for definitive exclusion of reverse causation. Second, the analytic sample was derived from a biomarker sub-sample of the CLHLS using a complete-case approach, and therefore some degree of selection bias cannot be excluded. In addition, because the biomarker sub-study was conducted in selected longevity areas rather than the full national CLHLS sample, the generalizability of our findings to the broader Chinese oldest-old population may be limited. Third, several LE8 components were operationalized using adapted definitions due to data availability constraints, which may have introduced measurement error. Specifically, diet and physical activity were constructed from available questionnaire items rather than the full AHA LE8 dietary and physical activity metrics. Similarly, HbA1c was not directly measured, and the blood glucose component was assigned using an HbA1c-equivalent value derived from fasting plasma glucose. These adaptations allowed the LE8 framework to be applied in the CLHLS biomarker sub-study but may limit direct comparability with studies using the original American Heart Association LE8 scoring algorithm. Finally, residual confounding may persist despite comprehensive multivariable adjustment. Future longitudinal studies are warranted to confirm these findings, clarify temporal relationships, and determine whether age-specific or function-oriented refinements of cardiovascular health metrics, particularly with respect to blood pressure assessment, may more accurately characterize disability risk in the oldest-old.

In conclusion, higher LE8 scores were associated with lower odds of ADL disability and reduced disability severity among Chinese adults aged ≥80 years. Among individual LE8 components, physical activity and diet showed the most prominent inverse associations with ADL disability, whereas the blood pressure component showed an unexpected pattern that should be interpreted cautiously in this age group. These findings support the potential value of LE8 as an integrated framework for characterizing functional vulnerability in the oldest-old. Further longitudinal studies are needed to confirm these associations and to determine whether age-specific refinements of cardiovascular health metrics may improve the assessment of disability risk in the oldest-old.

## Data Availability

The original contributions presented in the study are included in the article/Supplementary Material, further inquiries can be directed to the corresponding authors.
