# Supplementary material for: Association between life’s essential 8 and activities of daily living disability in Chinese adults aged 80 years and older: a cross-sectional study from the CLHLS
Source: Front Aging. 2026 Jul 10;7:1845642. doi: 10.3389/fragi.2026.1845642 (PMC13395931; doi:10.3389/fragi.2026.1845642)
Supplement: Supplementary file 1 [file Supplementaryfile1.docx]

**Association between Life’s Essential 8 With Activities and Daily Living Disability in Chinese Adults Aged 80 Years and Older: A Cross-Sectional Study from the CLHLS**

**Supplementary statistical methods**

**Study sample and outcomes**

The analytic sample was restricted to participants from the 2014 Chinese Longitudinal Healthy Longevity Survey (CLHLS) biomarker sub-study who were aged ≥80 years, had sufficient information to construct Life’s Essential 8 (LE8), and had available activities of daily living (ADL) data.

ADL disability was defined as the presence of any limitation in six basic ADL domains: bathing, dressing, toileting, indoor transferring, continence, and eating. For binary analyses, participants with ≥1 limitation were classified as having ADL disability. For severity analyses, an ordinal ADL outcome was created with three levels: 0 limitations, 1 limitation, and ≥2 limitations. Participants with ≥2 limitations were considered to have severe ADL disability.

**Construction of Life’s Essential 8**

LE8 was constructed using eight components: diet, physical activity, nicotine exposure, sleep health, body mass index, blood lipids, blood glucose, and blood pressure. Each component was scored from 0 to 100, with higher scores indicating more favorable cardiovascular health. The overall LE8 score was calculated as the mean of the eight component scores.

Nicotine exposure, sleep health, body mass index, blood lipids, and blood pressure were operationalized according to the American Heart Association LE8 framework when corresponding CLHLS variables were available. Diet and physical activity were adapted using available CLHLS questionnaire items because the original American Heart Association dietary and physical activity metrics could not be fully reconstructed. Following previously published CLHLS-based approaches, diet and physical activity scores were initially constructed using available questionnaire information and then linearly transformed to a 0–100 scale to align with the LE8 scoring framework.

For the blood glucose component, HbA1c was not available in the CLHLS biomarker data. Therefore, fasting plasma glucose was used to derive an HbA1c-equivalent value based on the published ADAG relationship:

HbA1c (%) = [eAG (mg/dL) + 46.7] / 28.7.

In this study, fasting plasma glucose was used as the available glucose biomarker to approximate this conversion. The derived HbA1c-equivalent value was used to assign the glucose component score according to American Heart Association LE8 glucose categories. Because this value was derived rather than directly measured, the glucose component should be interpreted as an adapted proxy measure. Detailed scoring criteria and original component distributions are presented in the Supplementary Tables.

**Baseline comparisons**

Continuous variables are presented as mean and standard deviation and were compared across LE8 tertiles using one-way analysis of variance. Categorical variables are presented as number and percentage and were compared using the χ² test.

**Primary regression models**

LE8 was modeled both categorically as tertiles and continuously per 10-point increase. Binary logistic regression was used for the outcome of any ADL disability. Proportional odds ordinal logistic regression was used for the three-level ADL disability severity outcome: 0 limitations, 1 limitation, and ≥2 limitations. The proportional odds assumption was evaluated using a likelihood-ratio test.

Three nested models were prespecified. Model 1 was unadjusted. Model 2 was adjusted for age and sex. Model 3 was additionally adjusted for economic status, education, residence, self-rated health, and marital status. Effect estimates are reported as odds ratios with 95% confidence intervals.

**Nonlinearity and visualization**

To explore potential nonlinearity, restricted cubic splines with five knots were fitted for continuous LE8 in the fully adjusted model. A Wald test was used to assess potential nonlinearity.

Model-based predicted probability curves were generated using the proportional odds model to visualize the predicted probabilities of any ADL disability and severe ADL disability across the range of LE8 scores. For visualization, covariates were held constant at their sample means for continuous variables or sample proportions for categorical variables.

**Component-level analyses**

To avoid overinterpretation of non-standard domain classifications, the revised analysis focused on the overall LE8 score and individual LE8 components. For exploratory component-level analyses, each LE8 component score was standardized, and odds ratios were estimated per 1-standard deviation increase in each component score. Components were analyzed in relation to binary ADL disability using multivariable logistic regression with the same covariate adjustment strategy as the primary analysis. Results were summarized using forest plots.

**Subgroup and interaction analyses**

Prespecified subgroup analyses were conducted by sex and age group. Age was categorized as 80–89 years and ≥90 years. Additional subgroup analyses were conducted according to chronic disease burden and frailty status to evaluate whether the association between LE8 and ADL disability differed by underlying health status.

Chronic disease burden was defined according to the number of available chronic conditions, including hypertension, diabetes, heart disease, and stroke/cerebrovascular disease. Participants were categorized into groups according to the number of chronic conditions.

Frailty status was defined using a frailty index based on the cumulative deficit model^1^. The frailty index was constructed as the proportion of accumulated health deficits across available variables, following previously established methods proposed by Rockwood and colleagues. Each deficit was coded between 0 and 1, with higher values indicating greater frailty burden. The frailty index was then used to classify participants into frailty subgroups for subgroup analyses. Details of the deficit variables included in the frailty index are provided in the Supplementary Tables.

Effect modification was evaluated by including multiplicative interaction terms between continuous LE8 and each subgroup indicator in the fully adjusted model. Interaction P-values were reported.

**Sensitivity analyses**

Robustness was assessed using the following sensitivity analyses:

1. additionally adjusting Model 3 for comorbidity burden, operationalized as the number of chronic conditions;
2. excluding participants with poor or very poor self-rated health;
3. excluding participants with pre-existing heart disease or stroke/cerebrovascular disease;
4. excluding bedridden participants;
5. additionally adjusting for co-residence status of the interviewee;
6. repeating analyses using severe ADL disability, defined as ≥2 limitations, as an alternative outcome definition.

As an additional sensitivity analysis, we repeated the component-level analysis for physical activity after excluding participants with poor or very poor self-rated health and after excluding bedridden participants. This analysis was conducted to assess whether the observed association between physical activity and ADL disability was primarily driven by participants with markedly poor perceived health or severe functional restriction.

**Exploratory analyses related to blood pressure**

Because the blood pressure component showed an association in an unexpected direction, we conducted exploratory analyses using original systolic and diastolic blood pressure values and repeated the overall LE8 analysis after removing the blood pressure component from the total score. These analyses were post hoc and were intended to assess whether the blood pressure component materially influenced the overall LE8–ADL association. Results were interpreted cautiously because of the cross-sectional design and the possibility of reverse causation, treatment effects, and age-related changes in blood pressure–outcome relationships.

**Supplementary references**

1 Searle SD, Mitnitski A, Gahbauer EA, Gill TM, Rockwood K. A standard procedure for creating a frailty index. *BMC Geriatrics*. 2008;8:24.

**Table S1 Components of physical activity score**

| Physical activity | Category | Score |
| --- | --- | --- |
| Regular exercise | Yes | 2 |
|  | No | 0 |
| Housework tasks |  |  |
|  | Almost everyday/Not every day, but at least once a week | 2 |
|  | Not every week, but at least once a month | 1 |
|  | Not every month, but sometimes/Never | 0 |
| Personal outdoor activities | Almost everyday/Not every day, but at least once a week | 2 |
|  | Not every week, but at least once a month | 1 |
|  | Not every month, but sometimes/ Never | 0 |
| Gardening | Almost everyday/Not every day, but at least once a week | 2 |
|  | Not every week, but at least once a month | 1 |
|  | Not every month, but sometimes/ Never | 0 |
| Rearing domestic animals/pets | Almost everyday/Not every day, but at least once a week | 2 |
|  | Not every week, but at least once a month | 1 |
|  | Not every month, but sometimes/ Never | 0 |
| Reading | Almost everyday/Not every day, but at least once a week | 2 |
|  | Not every week, but at least once a month | 1 |
|  | Not every month, but sometimes/ Never | 0 |
| Playing cards/mahjong | Almost everyday/Not every day, but at least once a week | 2 |
|  | Not every week, but at least once a month | 1 |
|  | Not every month, but sometimes/ Never | 0 |
| Watching TV/ listening to the radio | Almost everyday/Not every day, but at least once a week | 2 |
|  | Not every week, but at least once a month | 1 |
|  | Not every month, but sometimes/ Never | 0 |
| Attending social activities | Almost everyday/Not every day, but at least once a week | 2 |
|  | Not every week, but at least once a month | 1 |
|  | Not every month, but sometimes/ Never | 0 |

Physical activity score ranged from 0 to 18, and subsequently converted to a scale from 0 to 100 by dividing the maximum possible score and multiply by 100 .

**Table S2 Components of diet intake score**

| Diet intake | Category | Score |
| --- | --- | --- |
| Fresh vegetables consumption | Everyday or almost everyday/Quite often | 2 |
|  | Occasionally | 1 |
|  | Rarely or never | 0 |
| Fresh fruit consumption | Everyday or almost everyday/Quite often | 2 |
|  | Occasionally | 1 |
|  | Rarely or never | 0 |
| Legumes consumption | Almost everyday/ Not every day, but at least once a week | 2 |
|  | Not every week, but at least once a month | 1 |
|  | Not every month, but occasionally/Rarely or never | 0 |
| Meat consumption | Almost everyday/ Not every day, but at least once a week | 2 |
|  | Not every week, but at least once a month | 1 |
|  | Not every month, but occasionally/Rarely or never | 0 |
| Egg consumption | Almost everyday/ Not every day, but at least once a week | 2 |
|  | Not every week, but at least once a month | 1 |
|  | Not every month, but occasionally/Rarely or never | 0 |
| Fish and seafood consumption | Almost everyday/ Not every day, but at least once a week | 2 |
|  | Not every week, but at least once a month | 1 |
|  | Not every month, but occasionally/Rarely or never | 0 |
| Salty vegetables consumption | Almost everyday/ Not every day, but at least once a week | 0 |
|  | Not every week, but at least once a month | 1 |
|  | Not every month, but occasionally/Rarely or never | 2 |
| Tea consumption | Almost everyday/ Not every day, but at least once a week | 2 |
|  | Not every week, but at least once a month | 1 |
|  | Not every month, but occasionally/Rarely or never | 0 |
| Garlic consumption | Almost everyday/ Not every day, but at least once a week | 2 |
|  | Not every week, but at least once a month | 1 |
|  | Not every month, but occasionally/Rarely or never | 0 |

Diet score ranged from 0 to 18, and subsequently converted to a scale from 0 to 100 by dividing the maximum possible score and multiply by 100 .

**Table S3 Baseline characteristics of the original Life’s Essential 8 components**

| Characteristic | Tertile 1 (n=303) | Tertile 2  (n= 312) | Tertile 3 (n=294) | P value |
| --- | --- | --- | --- | --- |
| **Sleep duration (h)** | 7.38 ± 2.61 | 7.63 ± 1.76 | 7.62 ± 1.38 | 0.204 |
| **BMI** | 22.02 ± 4.14 | 21.07 ± 3.61 | 20.52 ± 2.85 | 0.000 |
| **SBP (mmHg)** | 154.50 ± 22.81 | 146.75 ± 23.20 | 135.72 ± 22.26 | 0.000 |
| **DBP (mmHg)** | 84.42 ± 12.76 | 80.23 ± 13.42 | 76.40 ± 12.58 | 0.000 |
| **Glucose (mmol/L)** | 5.40 ± 2.18 | 5.03 ± 0.87 | 4.87 ± 0.77 | 0.000 |
| **Non-HDL (mg/dL)** | 141.24 ± 39.44 | 129.46 ± 40.23 | 118.21 ± 28.65 | 0.000 |
| **Smoke (n,%)** |  |  |  | 0.000 |
| Never | 197 (65.0) | 259 (83.0) | 270 (91.8) |  |
| Current | 71 (23.4) | 35 (11.2) | 11 (3.7) |  |
| Former | 35 (11.6) | 18 (5.8) | 13 (4.4) |  |
| **Physical activity (n,%)** |  |  |  |  |
| Regular exercise |  |  |  | 0.002 |
| No | 270 (89.1%) | 277 (88.8%) | 236 (80.3%) |  |
| Yes | 33 (10.9%) | 35 (11.2%) | 58 (19.7%) |  |
| Housework tasks |  |  |  | 0.000 |
| Rarely or never | 150 (49.5%) | 155 (49.7%) | 90 (30.6%) |  |
| Occasionally | 9 (3.0%) | 5 (1.6%) | 5 (1.7%) |  |
| Almost every day | 144 (47.5%) | 152 (48.7%) | 199 (67.7%) |  |
| Personal outdoor activities |  |  |  | 0.000 |
| Rarely or never | 280 (92.4%) | 274 (87.8%) | 230 (78.2%) |  |
| Occasionally | 4 (1.3%) | 6 (1.9%) | 10 (3.4%) |  |
| Almost every day | 19 (6.3%) | 32 (10.3%) | 54 (18.4%) |  |
| Gardening |  |  |  | 0.000 |
| Rarely or never | 182 (60.1%) | 153 (49.0%) | 94 (32.0%) |  |
| Occasionally | 17 (5.6%) | 11 (3.5%) | 13 (4.4%) |  |
| Almost every day | 104 (34.3%) | 148 (47.4%) | 187 (63.6%) |  |
| Reading |  |  |  | 0.002 |
| Rarely or never | 287 (94.7%) | 287 (92.0%) | 251 (85.4%) |  |
| Occasionally | 3 (1.0%) | 4 (1.3%) | 6 (2.0%) |  |
| Almost every day | 13 (4.3%) | 21 (6.7%) | 37 (12.6%) |  |
| Rearing domestic animals/pets |  |  |  | 0.000 |
| Rarely or never | 252 (83.2%) | 243 (77.9%) | 203 (69.0%) |  |
| Occasionally | 7 (2.3%) | 3 (1.0%) | 5 (1.7%) |  |
| Almost every day | 44 (14.5%) | 66 (21.2%) | 86 (29.3%) |  |
| Playing cards/mahjong |  |  |  | 0.106 |
| Rarely or never | 280 (92.4%) | 278 (89.1%) | 251 (85.4%) |  |
| Occasionally | 4 (1.3%) | 7 (2.2%) | 8 (2.7%) |  |
| Almost every day | 19 (6.3%) | 27 (8.7%) | 35 (11.9%) |  |
| Watching TV/listening to the radio |  |  |  | 0.000 |
| Rarely or never | 151 (49.8%) | 131 (42.0%) | 75 (25.5%) |  |
| Occasionally | 14 (4.6%) | 18 (5.8%) | 12 (4.1%) |  |
| Almost every day | 138 (45.5%) | 163 (52.2%) | 207 (70.4%) |  |
| Attending social activities |  |  |  | 0.008 |
| Rarely or never | 296 (97.7%) | 305 (97.8%) | 278 (94.6%) |  |
| Occasionally | 4 (1.3%) | 4 (1.3%) | 2 (0.7%) |  |
| Almost every day | 3 (1.0%) | 3 (1.0%) | 14 (4.8%) |  |
| **Diet intake (n,%)** |  |  |  |  |
| Fresh fruit consumption |  |  |  | 0.000 |
| Rarely or never | 90 (29.7%) | 58 (18.6%) | 32 (10.9%) |  |
| Occasionally | 110 (36.3%) | 122 (39.1%) | 101 (34.4%) |  |
| Almost every day | 103 (34.0%) | 132 (42.3%) | 161 (54.8%) |  |
| Fresh vegetables consumption |  |  |  | 0.000 |
| Rarely or never | 17 (5.6%) | 10 (3.2%) | 1 (0.3%) |  |
| Occasionally | 42 (13.9%) | 36 (11.5%) | 23 (7.8%) |  |
| Almost every day | 244 (80.5%) | 266 (85.3%) | 270 (91.8%) |  |
| Meat consumption |  |  |  | 0.000 |
| Rarely or never | 68 (22.4%) | 41 (13.1%) | 16 (5.4%) |  |
| Occasionally | 33 (10.9%) | 25 (8.0%) | 20 (6.8%) |  |
| Almost every day | 202 (66.7%) | 246 (78.8%) | 258 (87.8%) |  |
| Fish and seafood consumption |  |  |  | 0.000 |
| Rarely or never | 120 (39.6%) | 81 (26.0%) | 42 (14.3%) |  |
| Occasionally | 59 (19.5%) | 51 (16.3%) | 46 (15.6%) |  |
| Almost every day | 124 (40.9%) | 180 (57.7%) | 206 (70.1%) |  |
| Eggs consumption |  |  |  | 0.000 |
| Rarely or never | 87 (28.7%) | 42 (13.5%) | 17 (5.8%) |  |
| Occasionally | 72 (23.8%) | 54 (17.3%) | 39 (13.3%) |  |
| Almost every day | 144 (47.5%) | 216 (69.2%) | 238 (81.0%) |  |
| Legumes consumption |  |  |  | 0.000 |
| Rarely or never | 108 (35.6%) | 62 (19.9%) | 32 (10.9%) |  |
| Occasionally | 77 (25.4%) | 91 (29.2%) | 75 (25.5%) |  |
| Almost every day | 118 (38.9%) | 159 (51.0%) | 187 (63.6%) |  |
| Tea consumption |  |  |  | 0.000 |
| Rarely or never | 253 (83.5%) | 244 (78.2%) | 192 (65.3%) |  |
| Occasionally | 11 (3.6%) | 10 (3.2%) | 10 (3.4%) |  |
| Almost every day | 39 (12.9%) | 58 (18.6%) | 92 (31.3%) |  |
| Garlic consumption |  |  |  | 0.000 |
| Rarely or never | 184 (60.7%) | 160 (51.3%) | 125 (42.5%) |  |
| Occasionally | 58 (19.1%) | 66 (21.2%) | 59 (20.1%) |  |
| Almost every day | 61 (20.1%) | 86 (27.6%) | 110 (37.4%) |  |
| Salty vegetables consumption |  |  |  | 0.008 |
| Rarely or never | 94 (31.0%) | 116 (37.2%) | 117 (39.8%) |  |
| Occasionally | 49 (16.2%) | 47 (15.1%) | 63 (21.4%) |  |
| Almost every day | 160 (52.8%) | 149 (47.8%) | 114 (38.8%) |  |

**Table S4. Distribution of ADL disability severity in the analytic sample**

| A. Collapsed categories (used in ordinal models) | |
| --- | --- |
| ADL disability category | n (%) |
| 0 | 777 (85.5) |
| 1 | 67 (7.4) |
| ≥2 | 65 (7.2) |
| B. Full distribution of number of ADL limitations | |
| Number of ADL limitations | n (%) |
| 0 | 777 (85.5) |
| 1 | 67 (7.4) |
| 2 | 11 (1.2) |
| 3 | 11 (1.2) |
| 4 | 12 (1.3) |
| 5 | 21 (2.3) |
| 6 | 10 (1.1) |

Abbreviations: ADL, activities of daily living; LE8, Life’s Essential 8.

Analytic sample: participants aged ≥80 years with complete LE8, ADL, and covariate data (N = 909).

**Table S5. Comparison of characteristics between included and excluded participants among adults aged ≥80 years with blood biomarker data**

| Characteristic | Excluded (n = 817) | Included (n = 909) | P value |
| --- | --- | --- | --- |
| Age, years | 90.91 (7.65) | 90.78 (7.64) | 0.314 |
| Male, n (%) | 319 (39.0) | 368 (40.5) | 0.542 |
| **Married, n (%)** | **184 (22.5)** | **254 (27.9)** | **0.010** |
| Economic status, n (%) |  |  | 0.220 |
| Rich | 81 (12.7) | 142 (15.6) |  |
| Fair | 643 (78.7) | 695 (76.5) |  |
| Poor | 70 (8.6) | 72 (7.9) |  |
| Self-rated health, n (%) |  |  | 0.061 |
| Good/very good | 377 (46.1) | 466 (51.3) |  |
| Fair | 341 (41.7) | 356 (39.1) |  |
| Poor/very poor | 99 (12.1) | 87 (9.6) |  |
| Education >5 years, n (%) | 50 (6.1) | 72 (7.9) | 0.145 |
| Urban residence, n (%) | 173 (21.2) | 188 (20.7) | 0.801 |
| Hypertension, n (%) | 231 (28.3) | 292 (32.1) | 0.082 |
| Heart disease, n (%) | 64 (7.8) | 95 (10.5) | 0.060 |
| Diabetes, n (%) | 13 (1.6) | 25 (2.8) | 0.101 |
| Stroke or cerebrovascular disease, n (%) | 43 (5.3) | 42 (4.6) | 0.538 |

**Table S6. Associations of individual LE8 components with ADL disability**

| LE8 component | SD | Model 1 OR (95% CI) | P value | Model 2 OR (95% CI) | P value | Model 3 OR (95% CI) | P value |
| --- | --- | --- | --- | --- | --- | --- | --- |
| Nicotine exposure score | 33.96 | 1.17  (0.95–1.45) | 0.129 | 1.00  (0.80–1.23) | 0.970 | 1.00  (0.81–1.24) | 0.996 |
| Sleep health score | 29.55 | 1.06  (0.89–1.27) | 0.517 | 1.11  (0.91–1.35) | 0.300 | 1.10  (0.91–1.34) | 0.328 |
| BMI score | 13.39 | 1.08  (0.90–1.28) | 0.405 | 1.01  (0.85–1.21) | 0.894 | 1.01  (0.85–1.21) | 0.873 |
| Blood pressure score | 30.66 | 1.23  (1.03–1.47) | 0.025 | 1.21  (1.00–1.45) | 0.047 | **1.22**  **(1.01–1.46)** | 0.039 |
| Physical activity score | 29.98 | 0.28  (0.20–0.38) | <0.001 | 0.32  (0.23–0.45) | <0.001 | **0.31**  **(0.22–0.44)** | <0.001 |
| Diet score | 27.03 | 0.76  (0.63–0.91) | 0.003 | 0.79  (0.65–0.96) | 0.017 | **0.78**  **(0.64–0.95)** | 0.013 |
| Blood lipids (non-HDL) score | 28.30 | 0.99  (0.82–1.19) | 0.909 | 0.94  (0.78–1.14) | 0.523 | 0.93  (0.77–1.13) | 0.489 |
| Blood glucose score | 19.88 | 1.00  (0.83–1.21) | 0.970 | 0.97  (0.79–1.19) | 0.749 | 0.96  (0.78–1.19) | 0.734 |

Note: Outcome: ADL disability (≥1 limitation). Effect estimates are expressed per 1-SD increase in each component score.

Model 1: unadjusted. Model 2: adjusted for age and sex. Model 3: additionally adjusted for economic status, education, residence, self-rated health and marital status.

**Table S7. Association of SBP and DBP with ADL disability per 10 mmHg increase**

| **Model** | **N** | **OR** | **95% CI** | **P value** |
| --- | --- | --- | --- | --- |
| **SBP** | | | | |
| Model 1 | 909 | 0.890 | 0.822–0.965 | 0.005 |
| Model 2 | 909 | 0.903 | 0.832–0.980 | 0.014 |
| Model 3 | 909 | 0.901 | **0.830–0.978** | 0.013 |
| **DBP** | | | | |
| Model 1 | 909 | 0.920 | 0.798–1.059 | 0.246 |
| Model 2 | 909 | 0.950 | 0.823–1.097 | 0.486 |
| Model 3 | 909 | 0.954 | 0.826–1.102 | 0.520 |

**Abbreviations: SBP,** systolic blood pressure; DBP, diastolic blood pressure; ADL, activities of daily living; OR, odds ratio; CI, confidence interval.

Model 1: unadjusted. Model 2: adjusted for age and sex. Model 3: additionally adjusted for economic status, education, residence, self-rated health and marital status.

**Table S8. Leave-BP-out analyses of the association between LE8 and ADL disability**

| Model | OR (95% CI) per 10-point increase | P value |
| --- | --- | --- |
| Binary ADL disability | | |
| Model 1 | 0.66 (0.55, 0.79) | <0.001 |
| Model 2 | 0.66 (0.54, 0.80) | <0.001 |
| Model 3 | **0.68 (0.56, 0.84)** | <0.001 |
| Ordinal ADL disability severity | | |
| Model 1 | 0.65 (0.54, 0.78) | <0.001 |
| Model 2 | 0.65 (0.53, 0.79) | <0.001 |
| Model 3 | **0.67 (0.55, 0.82)** | <0.001 |

**Table S9. Subgroup analyses for the association between LE8 and ADL disability (≥1 limitation)**

| Subgroup | N | ADL disability cases | OR per 10-point increase (95% CI) | P value | P for interaction |
| --- | --- | --- | --- | --- | --- |
| Sex |  |  |  |  |  |
| Male | 368 | 35 | 0.62 (0.46–0.83) | 0.002 | **0.114** |
| Female | 541 | 97 | 0.82 (0.64–1.06) | 0.131 |  |
| Age group |  |  |  |  |  |
| 80–89 | 450 | 34 | 0.68 (0.49–0.95) | 0.024 | **0.839** |
| ≥90 | 459 | 98 | 0.76 (0.58–0.98) | 0.036 |  |
| Chronic disease burden |  |  |  |  |  |
| 0-1 | 810 | 113 | 0.73 (0.58-0.93) | 0.009 | **0.536** |
| ≥2 | 99 | 19 | 0.66 (0.32-1.36) | 0.260 |  |
| Frailty status^*^ |  |  |  |  |  |
| Frail | 150 | 60 | 0.74 (0.47-1.18) |  | **0.672** |
| Non-frail | 695 | 63 | 0.68 (0.52-0.90) |  |  |

Note: Model: adjusted for age, sex, economic status, education, residence, self-rated health and marital status (excluding the stratification variable where applicable).

Effect estimates are expressed per 10-point increase in LE8 score.

P for interaction was obtained by adding a multiplicative interaction term (LE8 per 10 points × subgroup) to the fully adjusted model.

* The binary FI variable was missing for 60 participants. After excluding participants with missing FI or covariate data, 849 participants were included in the fully adjusted FI subgroup analysis.

**Table S10. Sensitivity analyses for the association between LE8 and severe ADL disability (aged ≥80 years)**

| **Outcome / Exposure** | **Model 1 OR (95% CI)** | **P** | **Model 2 OR (95% CI)** | **P** | **Model 3 OR (95% CI)** | **P** |
| --- | --- | --- | --- | --- | --- | --- |
| **Outcome: severe ADL disability (≥ 2 item) vs non-severe disability (binary logistic regression)** | | | | | | |
| LE8 (per +10 points) | 0.73 (0.57–0.93) | 0.012 | 0.74 (0.56–0.98) | 0.033 | **0.74 (0.56–0.97)** | 0.029 |
| LE8 tertiles: Middle vs Worst | 0.86 (0.49–1.50) | 0.588 | 0.81 (0.45–1.46) | 0.483 | 0.80 (0.45–1.44) | 0.455 |
| LE8 tertiles: Best vs Worst | 0.42 (0.21–0.84) | 0.014 | 0.46 (0.22–0.95) | 0.035 | **0.45 (0.22–0.93)** | 0.031 |

Note: Model 1: unadjusted. Model 2: adjusted for age and sex. Model 3: additionally adjusted for economic status, education, residence, self-rated health and marital status.

**Table S11. Sensitivity analyses for the association between LE8 and ADL disability (aged ≥80 years) (Additional adjustment for** **number of chronic conditions)**

| **Outcome / Exposure** | **Model 1 OR (95% CI)** | **P** | **Model 2 OR (95% CI)** | **P** | **Model 3 OR (95% CI)** | **P** |
| --- | --- | --- | --- | --- | --- | --- |
| **Outcome: ADL disability (≥ 1 item) vs no disability (binary logistic regression)** | | | | | | |
| LE8 (per +10 points) | 0.73 (0.61–0.88) | <0.001 | 0.74 (0.61–0.90) | 0.002 | **0.76 (0.62–0.94)** | 0.011 |
| LE8 tertiles: Middle vs Worst | 0.84 (0.55–1.28) | 0.419 | 0.80 (0.52–1.25) | 0.327 | 0.86 (0.55–1.35) | 0.519 |
| LE8 tertiles: Best vs Worst | 0.47 (0.29–0.77) | 0.003 | 0.51 (0.31–0.84) | 0.009 | **0.55 (0.33–0.94)** | 0.027 |
| **Outcome: ADL disability severity (0=no; 1=mild; 2=severe [≥2 items]) (ordinal logistic regression)** | | | | | | |
| LE8 (per +10 points) | 0.73 (0.60–0.88) | 0.001 | 0.73 (0.60–0.90) | 0.003 | **0.76 (0.62–0.94)** | 0.011 |
| LE8 tertiles: Middle vs Worst | 0.84 (0.55–1.28) | 0.420 | 0.80 (0.52–1.24) | 0.322 | 0.86 (0.55–1.34) | 0.508 |
| LE8 tertiles: Best vs Worst | 0.47 (0.29–0.77) | 0.002 | 0.50 (0.30–0.83) | 0.007 | **0.55 (0.33–0.91)** | 0.021 |

Note: Model 1: unadjusted. Model 2: adjusted for age and sex. Model 3 additionally adjusted for economic status, education, residence, self-rated health and marital status. Proportional odds assumption (LR test): *P* all > 0.05.

**Table S12. Sensitivity analyses for the association between LE8 and ADL disability (aged ≥80 years) (Excluding participants with poor or very poor self-rated health)**

| **Outcome / Exposure** | **Model 1 OR (95% CI)** | **P** | **Model 2 OR (95% CI)** | **P** | **Model 3 OR (95% CI)** | **P** |
| --- | --- | --- | --- | --- | --- | --- |
| **Outcome: ADL disability (≥ 1 item) vs no disability (binary logistic regression)** | | | | | | |
| LE8 (per +10 points) | 0.69 (0.56, 0.86) | <0.001 | 0.70 (0.56, 0.88) | 0.002 | **0.71 (0.56, 0.89)** | 0.003 |
| LE8 tertiles: Middle vs Worst | 0.62 (0.38, 1.01) | 0.053 | 0.62 (0.37, 1.02) | 0.058 | 0.63 (0.38, 1.04) | 0.072 |
| LE8 tertiles: Best vs Worst | 0.43 (0.25, 0.72) | 0.002 | 0.46 (0.27, 0.79) | 0.005 | **0.48 (0.28, 0.83)** | 0.009 |
| **Outcome: ADL disability severity (0=no; 1=mild; 2=severe [≥2 items]) (ordinal logistic regression)** | | | | | | |
| LE8 (per +10 points) | 0.69 (0.55, 0.85) | <0.001 | 0.69 (0.55, 0.87) | 0.002 | **0.70 (0.55, 0.88)** | 0.002 |
| LE8 tertiles: Middle vs Worst | 0.62 (0.38, 1.00) | 0.050 | 0.61 (0.37, 1.00) | 0.049 | 0.62 (0.37, 1.02) | 0.059 |
| LE8 tertiles: Best vs Worst | 0.42 (0.25, 0.71) | 0.001 | 0.45 (0.26, 0.77) | 0.004 | **0.46 (0.27, 0.80)** | 0.006 |

Note: Model 1: unadjusted. Model 2: adjusted for age and sex. Model 3 additionally adjusted for economic status, education, residence, self-rated health and marital status. Proportional odds assumption (LR test): *P* all > 0.05.

**Table S13. Sensitivity analyses for the association between LE8 and ADL disability (aged ≥80 years) (****Excluding participants with pre-existing heart disease or stroke/cerebrovascular disease)**

| **Outcome / Exposure** | **Model 1 OR (95% CI)** | **P** | **Model 2 OR (95% CI)** | **P** | **Model 3 OR (95% CI)** | **P** |
| --- | --- | --- | --- | --- | --- | --- |
| **Outcome: ADL disability (≥ 1 item) vs no disability (binary logistic regression)** | | | | | | |
| LE8 (per +10 points) | 0.68 (0.55, 0.84) | <0.001 | 0.69 (0.55, 0.87) | 0.001 | **0.73 (0.57, 0.92)** | 0.007 |
| LE8 tertiles: Middle vs Worst | 0.73 (0.46, 1.16) | 0.184 | 0.73 (0.45, 1.18) | 0.198 | 0.72 (0.44, 1.18) | 0.197 |
| LE8 tertiles: Best vs Worst | 0.42 (0.24, 0.72) | 0.002 | 0.46 (0.26, 0.80) | 0.006 | **0.52 (0.30, 0.93)** | 0.026 |
| **Outcome: ADL disability severity (0=no; 1=mild; 2=severe [≥2 items]) (ordinal logistic regression)** | | | | | | |
| LE8 (per +10 points) | 0.68 (0.55, 0.83) | <0.001 | 0.69 (0.55, 0.86) | <0.001 | **0.72 (0.57, 0.90)** | 0.005 |
| LE8 tertiles: Middle vs Worst | 0.73 (0.46, 1.15) | 0.177 | 0.73 (0.46, 1.18) | 0.198 | 0.71 (0.44, 1.16) | 0.170 |
| LE8 tertiles: Best vs Worst | 0.41 (0.24, 0.70) | 0.001 | 0.44 (0.25, 0.78) | 0.004 | **0.51 (0.29, 0.89)** | 0.019 |

Note: Model 1: unadjusted. Model 2: adjusted for age and sex. Model 3 additionally adjusted for economic status, education, residence, self-rated health and marital status. Proportional odds assumption (LR test): *P* all > 0.05.

**Table S14. Sensitivity analyses for the association between LE8 and ADL disability (aged ≥80 years) (Excluding bedridden participants)**

| **Outcome / Exposure** | **Model 1 OR (95% CI)** | **P** | **Model 2 OR (95% CI)** | **P** | **Model 3 OR (95% CI)** | **P** |
| --- | --- | --- | --- | --- | --- | --- |
| **Outcome: ADL disability (≥ 1 item) vs no disability (binary logistic regression)** | | | | | | |
| LE8 (per +10 points) | 0.73 (0.61–0.88) | <0.001 | 0.74 (0.61–0.90) | 0.002 | **0.75 (0.60–0.93)** | 0.010 |
| LE8 tertiles: Middle vs Worst | 0.84 (0.55–1.28) | 0.419 | 0.80 (0.52–1.25) | 0.327 | 0.72 (0.46–1.15) | 0.172 |
| LE8 tertiles: Best vs Worst | 0.47 (0.29–0.77) | 0.003 | 0.51 (0.31–0.84) | 0.009 | **0.55 (0.33–0.92)** | 0.024 |
| **Outcome: ADL disability severity (0=no; 1=mild; 2=severe [≥2 items]) (ordinal logistic regression)** | | | | | | |
| LE8 (per +10 points) | 0.73 (0.60–0.88) | 0.001 | 0.73 (0.60–0.90) | 0.003 | **0.75 (0.61–0.93)** | 0.009 |
| LE8 tertiles: Middle vs Worst | 0.84 (0.55–1.28) | 0.420 | 0.80 (0.52–1.24) | 0.322 | 0.70 (0.45–1.11) | 0.133 |
| LE8 tertiles: Best vs Worst | 0.47 (0.29–0.77) | 0.002 | 0.50 (0.30–0.83) | 0.007 | **0.54 (0.32–0.90)** | 0.019 |

Note: Model 1: unadjusted. Model 2: adjusted for age and sex. Model 3 additionally adjusted for economic status, education, residence, self-rated health and marital status. Proportional odds assumption (LR test): *P* all > 0.05.

**Table S15. Sensitivity analyses for the association between LE8 and ADL disability (aged ≥80 years) (Additional adjustment for co-residence of interviewee)**

| **Outcome / Exposure** | **Model 1 OR (95% CI)** | **P** | **Model 2 OR (95% CI)** | **P** | **Model 3 OR (95% CI)** | **P** |
| --- | --- | --- | --- | --- | --- | --- |
| **Outcome: ADL disability (≥ 1 item) vs no disability (binary logistic regression)** | | | | | | |
| LE8 (per +10 points) | 0.73 (0.61–0.88) | <0.001 | 0.74 (0.61–0.90) | 0.002 | **0.74 (0.60–0.92)** | 0.007 |
| LE8 tertiles: Middle vs Worst | 0.84 (0.55–1.28) | 0.419 | 0.80 (0.52–1.25) | 0.327 | 0.72 (0.46–1.14) | 0.164 |
| LE8 tertiles: Best vs Worst | 0.47 (0.29–0.77) | 0.003 | 0.51 (0.31–0.84) | 0.009 | **0.53 (0.32–0.89)** | 0.017 |
| **Outcome: ADL disability severity (0=no; 1=mild; 2=severe [≥2 items]) (ordinal logistic regression)** | | | | | | |
| LE8 (per +10 points) | 0.73 (0.60–0.88) | 0.001 | 0.73 (0.60–0.90) | 0.003 | **0.74 (0.60–0.92)** | 0.006 |
| LE8 tertiles: Middle vs Worst | 0.84 (0.55–1.28) | 0.420 | 0.80 (0.52–1.24) | 0.322 | 0.71 (0.45–1.11) | 0.136 |
| LE8 tertiles: Best vs Worst | 0.47 (0.29–0.77) | 0.002 | 0.50 (0.30–0.83) | 0.007 | **0.52 (0.31–0.88)** | 0.014 |

Note: Model 1: unadjusted. Model 2: adjusted for age and sex. Model 3 additionally adjusted for economic status, education, residence, self-rated health and marital status. Proportional odds assumption (LR test): *P* all > 0.05.

**Table S16. Association of the physical activity component with ADL disability after excluding participants with poor or very poor self-rated health**

| Model | OR | 95% CI | P value |
| --- | --- | --- | --- |
| Model 1 | 0.34 | 0.26–0.45 | <0.001 |
| Model 2 | 0.39 | 0.30–0.53 | <0.001 |
| Model 3 | **0.39** | **0.29–0.52** | <0.001 |

Note: The physical activity component was analyzed per 1-SD increase. Model 1 was unadjusted. Model 2 was adjusted for age and sex. Model 3 was adjusted for age, sex, education, residence, marital status, self-rated health, and economic status. The analytic sample included 822 participants.

**Table S17. Association of the physical activity component with ADL disability after excluding bedridden participants**

| Model | OR | 95% CI | P value |
| --- | --- | --- | --- |
| Model 1 | 0.28 | 0.21–0.37 | <0.001 |
| Model 2 | 0.33 | 0.24–0.43 | <0.001 |
| Model 3 | **0.34** | **0.25–0.46** | <0.001 |

Note: The physical activity component was analyzed per 1-SD increase. Model 1 was unadjusted. Model 2 was adjusted for age and sex. Model 3 was adjusted for age, sex, education, residence, marital status, self-rated health, and economic status. The analytic sample included 897 participants.

**Excluded: missing ADL data (n = 57)**

**Excluded: incomplete LE8 components (n = 760)**

**Excluded: age < 80 (n = 2454)**

**Excluded: no blood biomarkers (n = 3012)**

**CLHLS 2014 baseline participants (N = 7192)**

**Age ≥ 80 years (N = 4738)**

**With blood biomarker data (N = 1726)**

**Able to construct LE 8 score (N = 966)**

**Final sample with ADL data (N = 909)**

**Figure S1. Study flow diagram chart.**


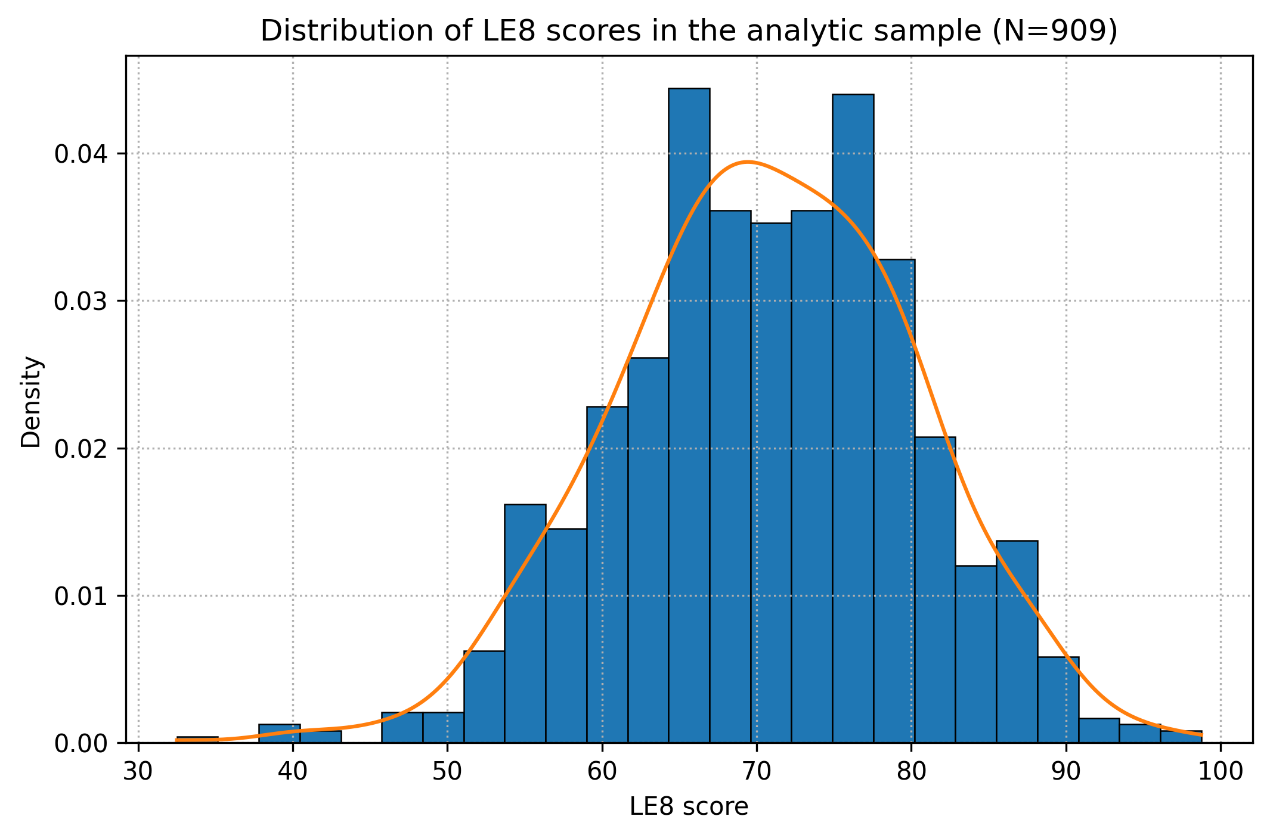


**Figure S2. Distribution of Life’s Essential 8 (LE8) scores in the analytic sample.**

The histogram shows the empirical distribution of LE8 scores among participants aged ≥80 years included in the primary analysis (N=909). The overlaid curve represents a kernel density estimate to visualize the smoothed distribution.


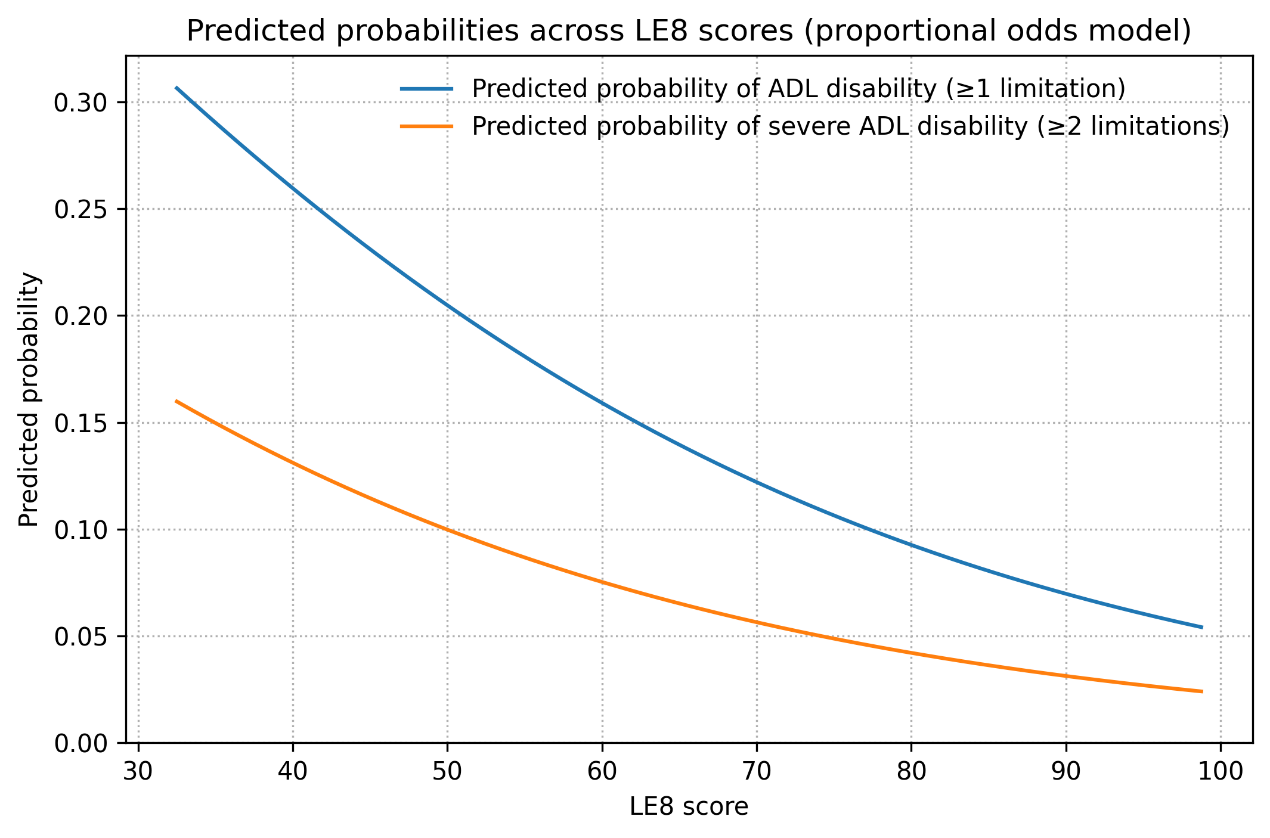


**Figure S3. Predicted probability curves for ADL disability across LE8 scores.**
The figure displays model-based predicted probabilities of (i) ADL disability (≥1 limitation) and (ii) severe ADL disability (≥2 limitations) across the range of Life’s Essential 8 (LE8) scores among participants aged ≥80 years. Predicted probabilities were derived from a proportional odds ordinal logistic regression model with ADL disability categorized as 0, 1, and ≥2 limitations.

**Note:** Predictions were obtained from the fully adjusted model (Model 3), including LE8 (per 10-point increase), age, sex, economic status, education, residence, and marital status. For visualization, covariates were held constant at their sample means (continuous) or sample proportions (binary). The y-axis shows the predicted probability of meeting each threshold (≥1 limitation and ≥2 limitations) at a given LE8 score.


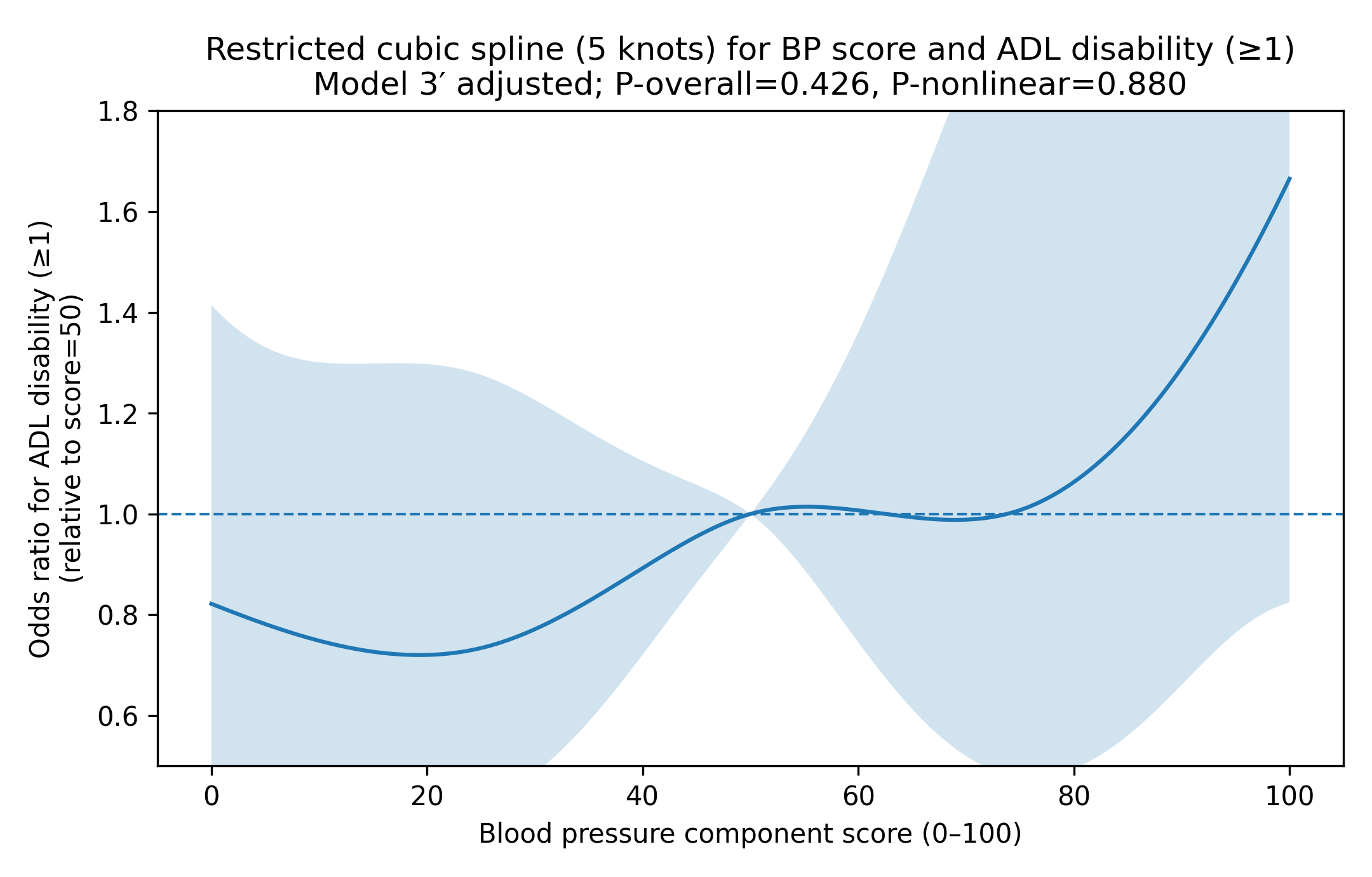


**Figure S4. Restricted cubic spline (5 knots) for BP component score and ADL disability (≥1)**

Knots were placed at BP score = 0, 25, 50, 75, and 100 (interior knots 25/50/75). Model 3′ adjusted for age, sex, education, residence, marital status, and economic status (N=909). Likelihood-ratio tests: P-overall = 0.426; P-nonlinear = 0.880. Odds ratios are shown relative to BP score = 50.


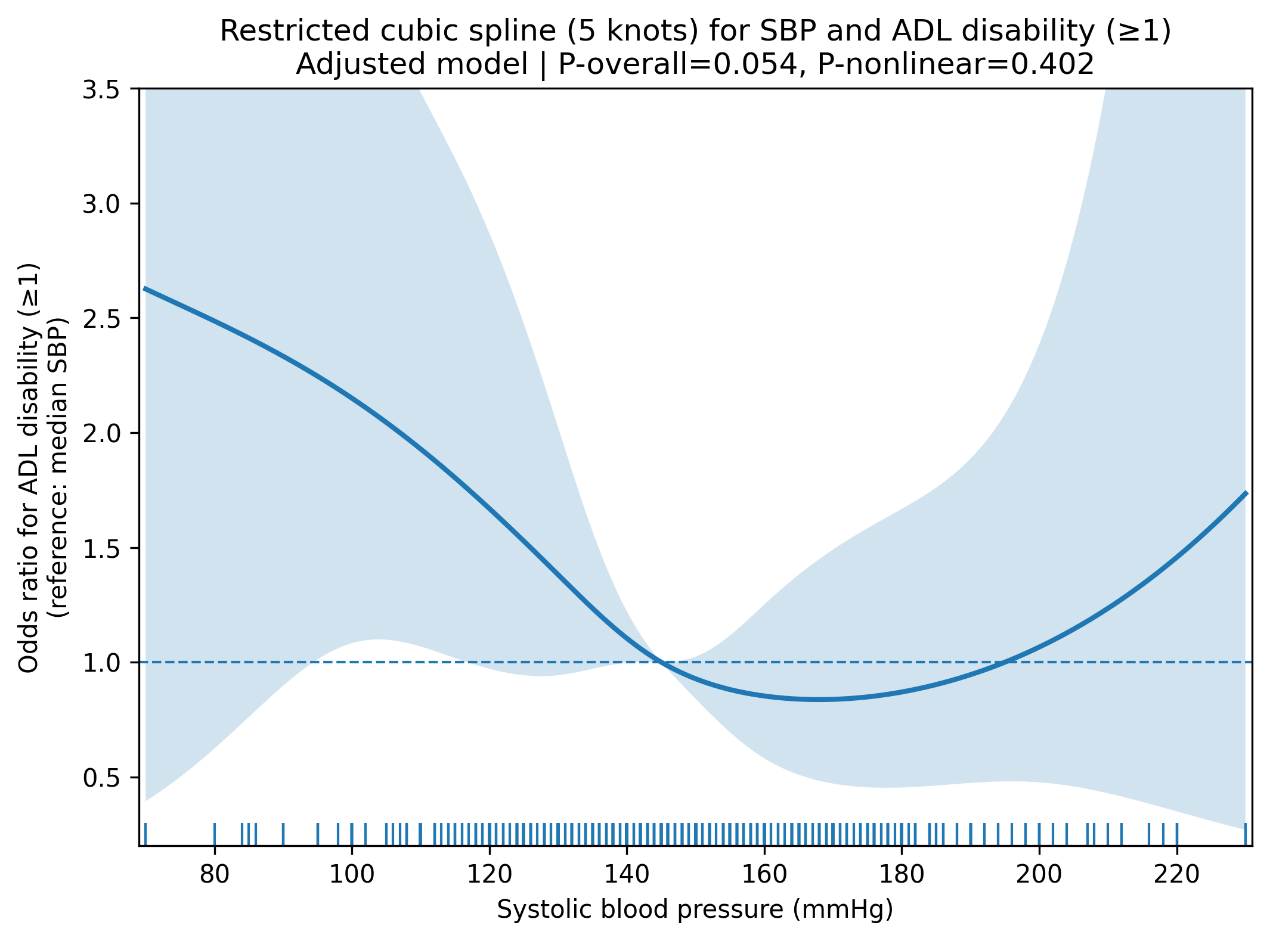


**Figure S5. Restricted cubic spline (5 knots) for systolic blood pressure (SBP) and ADL disability (≥1).** The curve shows adjusted odds ratios and 95% confidence intervals for ADL disability across the observed SBP range, with the median SBP (145 mmHg) used as the reference. The multivariable model was adjusted for age, sex, marital status, economic status, education, self-rated health, and residence. Tick marks on the x-axis indicate the distribution of SBP values.


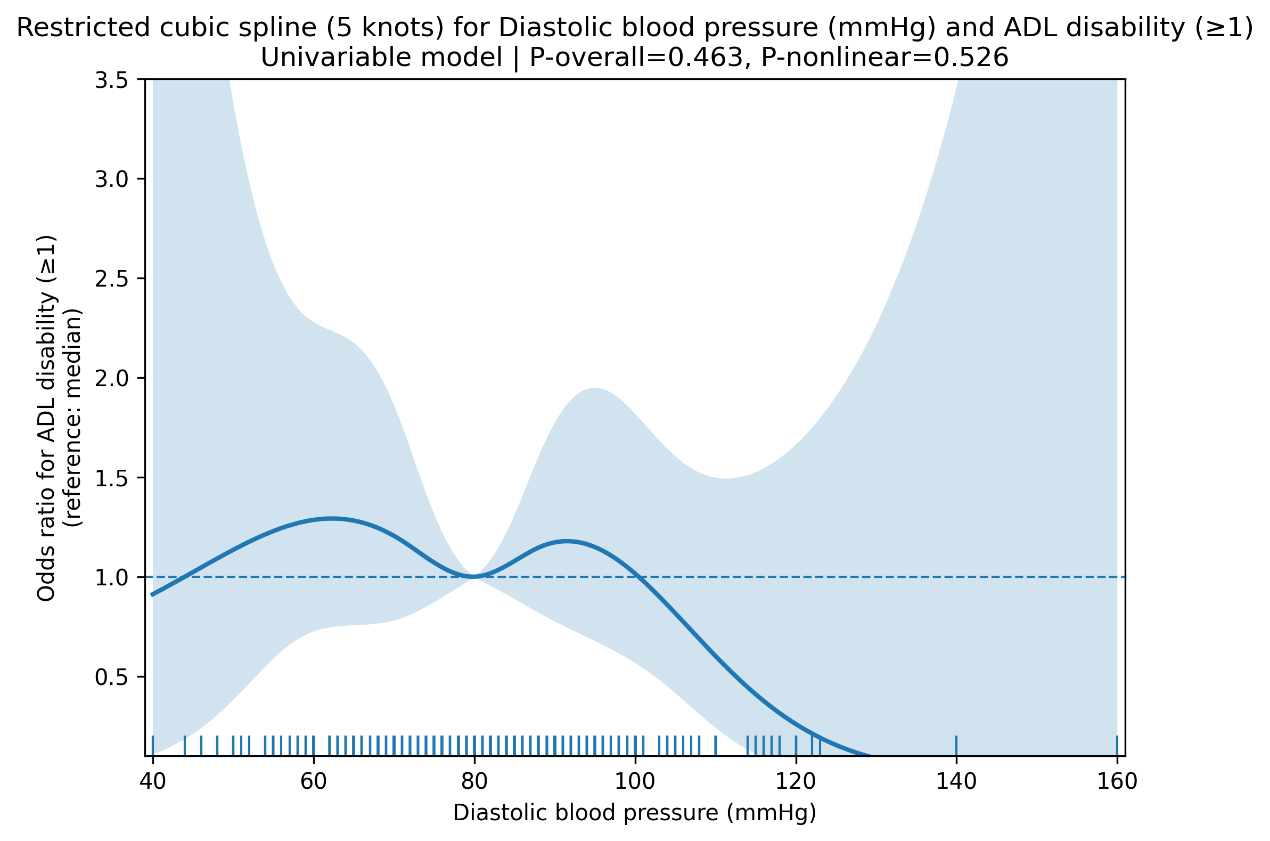


**Figure S6**. Restricted cubic spline analysis of diastolic blood pressure (DBP) and ADL disability (≥1). The solid line represents the adjusted odds ratio for ADL disability across the observed range of DBP, with the median DBP (80 mmHg) as the reference. The multivariable model was adjusted for age, sex, marital status, economic status, education, self-rated health, and residence. Tick marks on the x-axis indicate the distribution of SBP values.
